# An Immune Panel Signature Predicts Prognosis of Lung Adenocarcinoma Patients and Correlates With Immune Microenvironment

**DOI:** 10.3389/fcell.2021.797984

**Published:** 2021-12-21

**Authors:** Yuan Zhou, Lu Tang, Yuqiao Chen, Youyu Zhang, Wei Zhuang

**Affiliations:** ^1^ Department of Thoracic Surgery, Xiangya Hospital of Central South University, Changsha, China; ^2^National Clinical Research Center for Geriatric Disorders, Xiangya Hospital, Central South University,Changsha, China; ^3^ Department of Anesthesiology, Xiangya Hospital of Central South University, Changsha, China

**Keywords:** lung adenocarcinoma, progression-free survival, immune panel sequencing, gene signature, metastasis

## Abstract

**Background:** Lung cancer, especially lung adenocarcinoma (LUAD) with high incidence, seriously endangers human life. The immune microenvironment is one of the malignant foundations of LUAD, but its impact at the molecular level is incompletely understood.

**Method:** A total of 34 LUAD samples from Xiangya Hospital were collected for immune oncology (IO) profiling. Univariate Cox analysis was performed to profile prognostic immune genes based on our immune panel sequencing data. The least absolute shrinkage and selection operator (LASSO) algorithm was applied to construct a risk signature. The cut-off threshold of risk score was determined using X-tile software. Kaplan–Meier survival curves and receiver operating characteristic (ROC) curves were employed to examine the performance of this risk signature for predicting prognosis. The immune infiltration was estimated using a single-sample gene set enrichment analysis (ssGSEA) algorithm.

**Result:** Thirty-seven immune genes were profiled to be significantly correlated with the progression-free survival (PFS) in our cohort. Among them, *BST2*, *KRT7*, *LAMP3*, *MPO*, *S100A8*, and *TRIM29* were selected to construct a risk signature. Patients with a higher risk score had a significantly shorter PFS (*p* = 0.007). Time-dependent ROC curves indicated that our risk signature had a robust performance in accurately predicting survival. Specifically, the 6-, 12-, and 18-month area under curve (AUC) was 0.800, 0.932, and 0.912, respectively. Furthermore, the risk signature was positively related to N stage, tumor stage, and tumor malignancy. These results were validated using two external cohorts. Finally, the risk signature was **s**ignificantly and uniquely correlated with abundance of neutrophil.

**Conclusion:** Our study revealed an immune panel-based signature that could predict the prognosis of LUAD patients and was associated with the infiltration of neutrophils.

## Introduction

Lung cancer is nowadays the leading cause of cancer-related morbidity and mortality worldwide, accounting for nearly 20% of cancer deaths (Bray et al., 2018). Approximately 85% of patients have a group of histological subtypes collectively termed as non-small cell lung cancer (NSCLC), of which lung adenocarcinoma (LUAD) and lung squamous cell carcinoma (LUSC) are the most common subtypes (Herbst et al., 2018). Since they are usually asymptomatic at an early stage, most lung cancers (61%) are diagnosed at stage III or IV, with only 21% at stage I. More importantly, advanced lung cancer confers an extremely poor prognosis. In brief, the 5-year relative survival rate for stage I patients is 57% and decreases to 29% for patients with stage III lung cancers (Miller et al., 20192019).

Although improvements in surgical techniques and chemoradiotherapy, as well as individualized treatment regimens with tyrosine kinase inhibitors (TKIs) as the mainstay, have led to inspiring clinical advances (Pao and Girard, 2011; Miller et al., 20192019), there are still some populations that exhibit limited responses or acquire resistance. In addition, previous studies have explored more factors to predict the patient's prognosis in a more accurate manner, such as vascular spreading (Gabor et al., 2004) and lymphatic spreading (Popper, 2016). But the prediction of metastasis as well as the patient's prognosis at the clinical level is far from satisfactory. Thus, how to predict patient prognosis (herein specifically refers to the metastasis of lung cancer) is an urgent issue that remains unaddressed for clinicians. Therefore, we need to focus on the biological process and intrinsic malignant basis of LUAD at the molecular level in the hope of establishing good prognostic indicators or targeting regimens.

In 2018, the immune landscape of cancer has been depicted by conducting a comprehensive immunogenomic analysis of more than 10,000 samples across 33 cancer types (Thorsson et al., 2018). Samples could be well clustered based on the immune profiles and exhibit different molecular profiles and distinct survivals. Since then, efforts have been devoted to analyzing tumor–immune cell interaction in specific malignancies at the transcriptional level. However, we noticed that although several studies have applied bioinformatics in the context of lung cancers, most of them focused on publicly available databases such as classical The Cancer Genome Atlas (TCGA) database, making the conclusions limited and ungeneralizable.

It is well known that cancer cells can functionally construct a tumor microenvironment (TME) by regulating the reprogramming of surrounding cells, which play a decisive role in tumor survival and progression. Immune cells are important components of TME and play a critical role in this process. Both innate immune cells (macrophages, neutrophils, dendritic cells, and natural killer cells) and adaptive immune cells (T and B cells) contribute to tumor progression within the TME context. Dialogs between cancer cells and surrounding immune infiltrates ultimately result in a complexed network that promotes tumor growth and metastasis (Hinshaw and Shevde, 2019). Particularly, the TME has largely affected the efficacy of immunotherapies based on immune checkpoint blockade (He et al., 2015; Sharma et al., 2017; Herbst et al., 2018), which highlights the importance and the urgent need of deeply understanding how TME orchestrates the therapeutic and prognostic outcomes. Therefore, we employed our sequencing data to employ the prognostic immune genes and construct a risk signature for prognosis prediction and potential therapy target.

## Materials and Methods

### Data Extraction

We enrolled 34 LUAD samples in our institute from December 2014 to December 2016. The patients who met the following inclusion criteria were included: 1) postoperative pathology confirmed advanced lung cancer, and the pathological stage was T1–4, N1–2, M0; 2) no neoadjuvant therapy was administrated before surgery; and 3) disease progression events such as local recurrence or distant metastasis occurred during postoperative follow-up. Finally, a total of 34 corresponding samples were collected, and these samples were subsequently sequenced by Genecast Biotechnology, Beijing, China. Specifically, RNA immune oncology (IO) profiling was performed to quantify 395 IO associated genes related to tumor markers, basic signaling pathways, tumor-specific antigens, immune responses, infiltrating immune cells, and housekeeping (HK) genes in human solid cancers (Supplementary Table S1).

All of the patients (or their family representatives) have signed written inform consent, and this study was approved by the Ethics Committee of Xiangya Hospital, Central South University. The clinical information of included samples is summarized in Supplementary Table S2.

RNA-seq data and clinical information were extracted for validation from TCGA (https://portal.gdc.cancer.gov/) and the Gene Expression Omnibus (GEO; https://www.ncbi.nlm.nih.gov/geo) databases. A total of 510 and 443 patients with LUAD were extracted from TCGA-LUAD and GSE68465 datasets (Shedden et al., 2008), respectively.

### Identification of Prognostic Immune Genes

Univariate Cox analysis was performed on immune genes obtained in our immune panel sequencing data to profile those with significant correlations with progression-free survival (PFS) of patients with LUAD.

### Construction and Validation of Risk Signature

The least absolute shrinkage and selection operator (LASSO) algorithm was applied to refine the most representative of the prognostic immune genes and assign them corresponding coefficients (Boyd et al., 2011). The genes at the smallest lambda value were included to build the signature. And the risk score for each individual in the training and validation cohorts was calculated using the following formula: $\;\mathrm{Risk}\;score=\sum_{I}^{n}\left(\beta_{i}\times x_{i}\right)$ . n was the number of genes. $x_{i}$ represented the mRNA expression level of each model gene, and $\beta_{i}$ meant the coefficient.

The cut-off threshold to divide patients into high-risk and low-risk groups was determined using X-tile software (Camp et al., 2004). Kaplan–Meier survival curves and time-dependent receiver operating characteristic (ROC) curves were employed to examine the performance of this risk signature for predicting prognosis. Furthermore, the relationship between risk score and clinicopathological features, including American Joint Committee on Cancer (AJCC) tumor stage and degree of tumor differentiation, was also assessed.

### Immune Infiltration

Gene set variation analysis (GSVA) analysis was conducted using the “GSVA” R package (Hänzelmann et al., 2013). The immune infiltration of multiple cell types was estimated using single-sample gene set enrichment analysis (ssGSEA) algorithm.

### Statistical Analysis

Data analysis and visualization were conducted using GraphPad Prism version 8.0.1 and R language version 3.6.3. The cut-off value for risk score was determined using X-tile software. Student's t-test and Wilcoxon test were used to compare the difference between two groups. One-way analysis of variance (ANOVA) test and Kruskal–Wallis test were used to compare the difference among more than two groups. Kaplan–Meier analysis was used for survival analysis. A p-value <0.05 was considered statistically significant.

## Results

### Immune Gene-Based Risk Signature Predicts Prognosis and Correlates With Malignant Clinical Features

**FIGURE 1:**
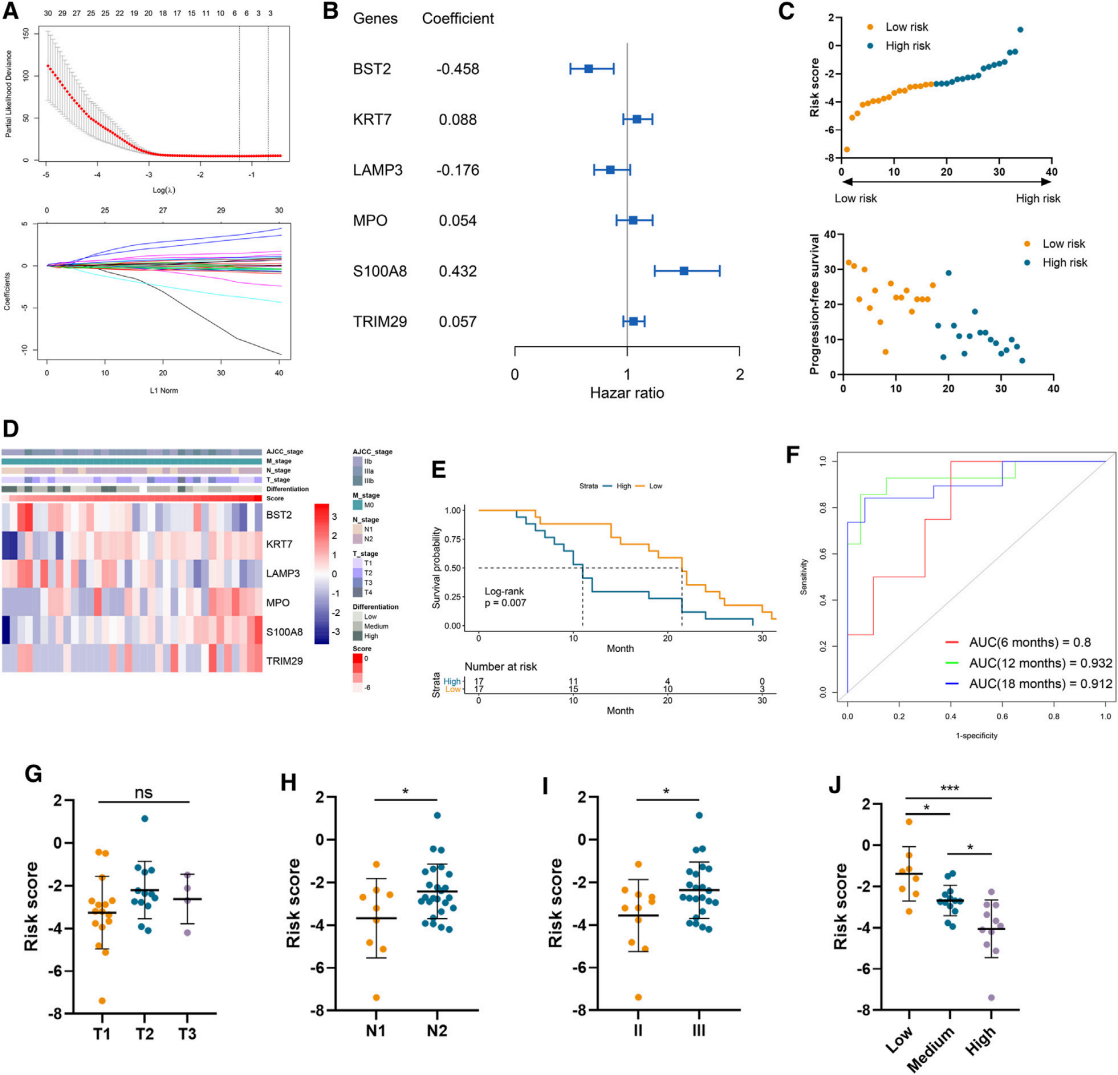
Construction of risk signature to predict the metastasis of lung adenocarcinoma. **(A)** LASSO analysis of prognostic genes with the minimum lambda value. **(B)** The coefficient of six genes to construct the risk signature. **(C)** Risk score of each sample was calculated based on the coefficient and expression of six genes. **(D)** Heatmap of the expression of six genes in samples with increasing risk score. **(E)** Kaplan–Meier analysis of high-risk and low-risk patients. **(F)** Time-dependent ROC analysis of risk score to predict the 6-, 12-, and 18-month survival. **(G–J)** The risk score in tumors with different T stages **(G)**, N stages **(H)**, AJCC stages **(I)**, and differentiation degree **(J)**.

A total of 37 genes were profiled to be significantly correlated with the PFS in our cohort involving 34 LUAD samples (Supplementary Table S3). And six of them, including bone marrow stromal cell antigen 2 (*BST2*), keratin 7 (*KRT7*), lysosomal associated membrane protein 3 (*LAMP3*), myeloperoxidase (*MPO*), S100 calcium binding protein A8 (*S100A8*), and tripartite motif containing 29 (*TRIM29*), were selected by the LASSO model to construct a risk signature (Figure 1A). Among them, *S100A8*, *TRIM29*, *MPO*, and *KRT7* were significant protective factors for LUAD patients, while LAMP3 and BST2 were risk factors in our cohort (Supplementary Figure S1). More importantly, these model genes exhibited consistent prognostic values in two external validation cohorts (Supplementary Figure S2). Their coefficients are displayed in Figure 1B and the distributions of risk scores and PFS status were illustrated in Figure 1C. The expression pattern of *BST2* as well as *LAMP3* was opposite to that of the risk score, and conversely, the same trend was observed for the four remaining model genes and the risk score (Figure 1D). Importantly, patient prognosis differed between the two risk groups, as patient with a higher risk score significantly demonstrated shorter PFS (Log-rank *p* = 0.007; Figure 1E). Furthermore, time-dependent ROC curves indicated that our risk signature had a robust performance in accurately predicting survival. Specifically, the 6-, 12-, and 18-month area under curve (AUC) was 0.800, 0.932, and 0.912, respectively (Figure 1F). Regarding clinical characteristics, risk score did not correlate with tumor T stage but was positively related to N stage, AJCC stage, and tumor malignancy (as negatively related to the degree of tumor differentiation). (Figures 1G–J).

### Validation of the Risk Signature Using Independent Cohorts With Large Sample Size

**FIGURE 2:**
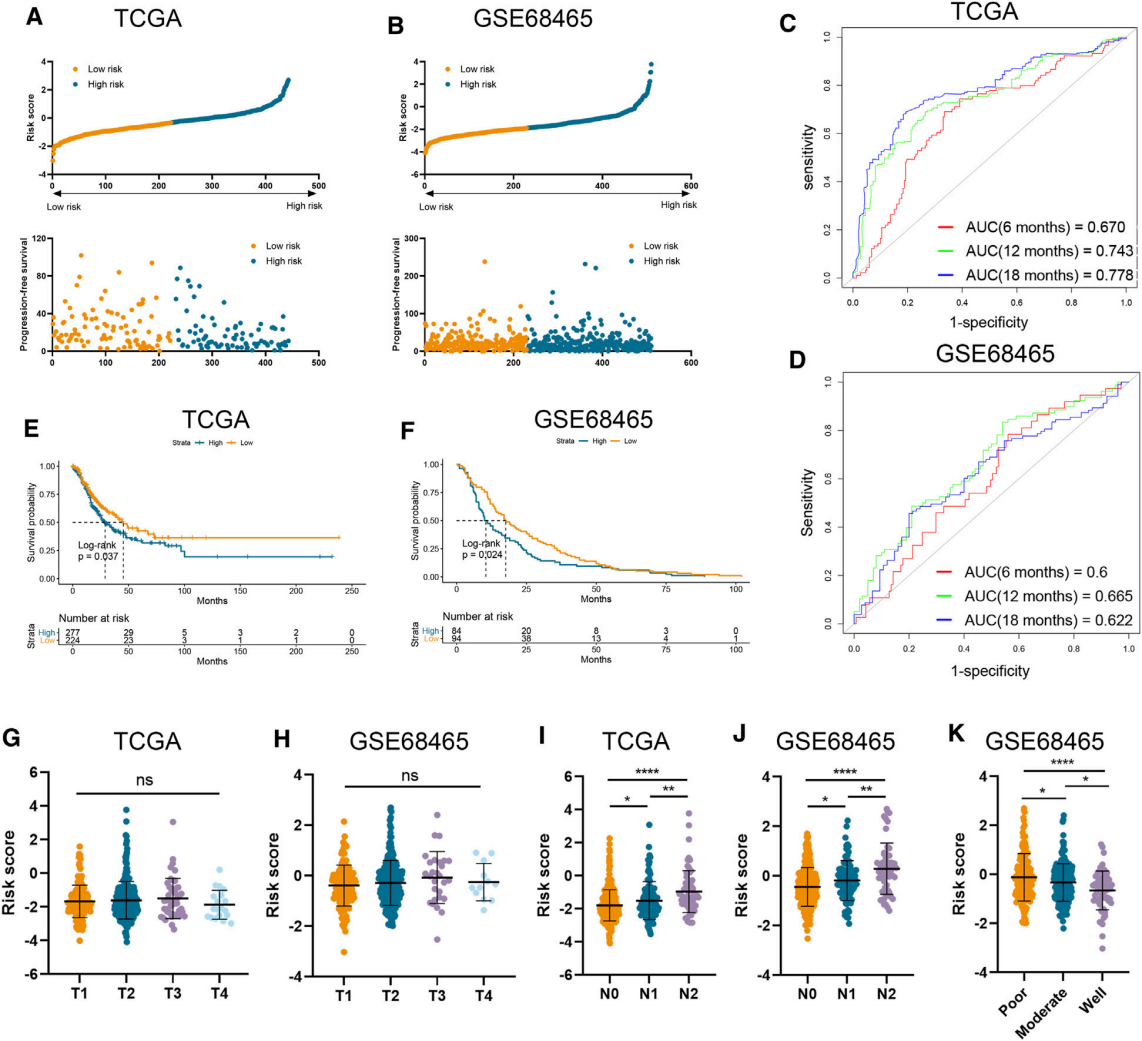
Validation of risk signature in two independent datasets. **(A,B)** Risk score was calculated in the TCGA **(A)** and GSE68465 **(B)** datasets. **(C,D)** Time-dependent ROC analysis of risk score to predict the 6-, 12-, and 18-month survival in TCGA **(C)** and GSE68465 **(D)** datasets. **(E,F)** Kaplan–Meier analysis of progression-free survival of high-risk and low-risk patients in TCGA **(E)** and GSE68465 **(F)** datasets. **(G–J)** The risk score in tumors with different T stages, and N stages in two datasets. **(K)** The risk score in tumors with different differentiation degrees in GSE68465.

The clinical and RNA sequencing data of TCGA-LUAD and GSE68465 cohorts with 510 and 443 LUAD patients were extracted for external validation. Similarly, patients with lower risk scores presented with longer PFS time than those with higher risk scores (Figures 2A, B). The performance of our risk signature in predicting prognosis was still excellent under validation, as the AUC was all above 0.6 in two cohorts (Figures 2C, D). Besides, Kaplan–Meier analyses also revealed significantly poorer outcomes in the high-risk groups (TCGA-LUAD log-rank *p* = 0.037; GSE68465 log-rank *p* = 0.024; Figures 2E, F). There was no significant correlation between risk signature and tumor T stage, which was consistent with the result in our training cohort (Figures 2G, H). And LUAD patients with higher AJCC N stage and lower degree of tumor differentiation had higher risk scores in both validation cohorts (Figures 2I–K).

### Risk Signature Correlates With Infiltrating Neutrophil in Tumor Microenvironment

**FIGURE 3:**
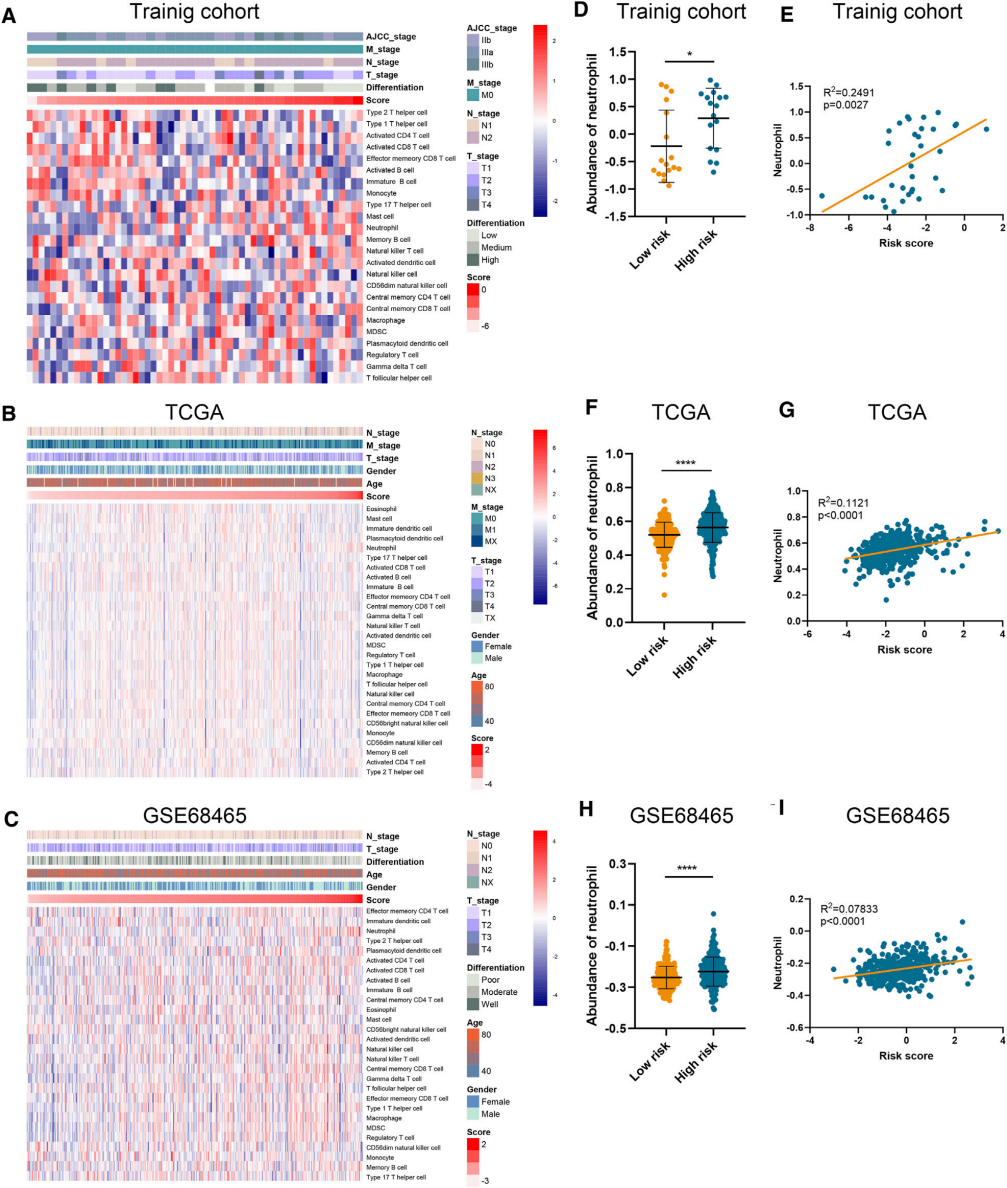
Association between risk score and immune infiltration. **(A–C)** The abundance of different immune cells in the training cohort **(A)**, TCGA **(B)**, and GSE68465 **(C)** datasets. **(D–I)** The abundance of neutrophil in high-risk and low-risk patients and its correlation with risk score in training cohort **(D,E)**, TCGA **(F,G)**, and GSE68465 **(H,I)** datasets.

Using ssGSEA algorithm, we obtained the abundances of 24 infiltrating cell types (Supplementary Table S4). Significant and unique correlation was detected between our risk signature and neutrophil (Figures 3A–C), as LUAD samples in the high-risk group possessed higher abundance of neutrophil than those in the low-risk group (Figures 3D, F, H). Moreover, the risk score was correlated with the abundance of neutrophil in the training cohort (*p* = 0.0027; Figure 3E), TCGA-LUAD cohort (*p* < 0.0001; Figure 3G), and GSE68465 cohort (*p* < 0.0001; Figure 3I). However, the risk score is not relevant to other types of immune cells.

## Discussion

Prodigious effort has been devoted to explore the underlying mechanisms of lung cancer at the molecular level, but the current understanding of TME and prognostic factors is still far from satisfactory. In this study, we first screened out the significant prognostic gene sets for LUAD in immune panel profiles and further investigated them. We constructed a novel risk signature using representative immune genes with significant prognostic value for LUAD. Gratifyingly, our six-gene risk signature allowed robust risk stratification as high-risk patients had a significantly worse prognosis (*p* = 0.007). And this model presented a predictive power with high specificity (AUC > 0.85). To our surprise, the trained model showed higher accuracy of 12- and 18-month survival prediction compared with the result from the 6-month survival prediction, and the superiority was kept when the prediction was done using the test sets. This is likely because the immune-related genetic characteristics have increasingly amplified with certain and consistent proportions during cancer progression.

Events for PFS refer to tumor progression (recurrence, enlargement, or metastasis) or death from any cause. And for LUAD, the length of PFS strongly depends on patients' metastatic events. Our risk signature was found to be significantly correlated with tumor N stage, AJCC stage, and tumor malignant property, which are independent predictors of lung cancer metastasis (Lim et al., 2018). This also, to some extent, explains the sound ability of our model in predicting metastasis.

A key finding in our study is that the established risk signature is significantly correlated with the abundance of neutrophil in all the cohorts. Tumor-associated neutrophils predict poor overall survival in many cancer types, with their location in the tumor and specific markers being important deferential determinants (Shaul and Fridlender, 2019). For example, massive expression of inflammation-related genes is transcriptionally activated by epigenetic remodeling in advanced clear cell renal cell carcinoma, which is related to metastasis in a neutrophil-dependent manner (Nishida et al., 2020). Meanwhile, neutrophils in lung mesenchyme are essential for breast cancer lung metastasis (Wculek and Malanchi, 2015; Li et al., 2020). More importantly, using single-cell RNA sequencing (scRNA-seq) to map tumor-infiltrating myeloid cells in non-small-cell lung cancer patients, neutrophils were found to be a key regulator of cancer growth across individuals and species (Zilionis et al., 2019). Combining these findings, we propose that our risk signature correlates with neutrophil population intrinsically and reflects the risk of metastasis in LUAD patients.

As for the functions of proteins encoded by signature genes, KRT7 is found to be tightly linked to cancer metastasis in colorectal cancer (Yu et al., 2017) and breast cancer (Chen et al., 2021). LAMP3 is implied to be involved in metastasis in esophageal squamous cell carcinoma (Huang et al., 2020) and uterine cervical cancer (Kanao et al., 2005) but in different pathways. Neutrophils play a vital role in both chemically mediating inflammatory response through MPO and biologically promoting metastasis during inflammation triggered by the environmental stimuli (Tang et al., 2020). Moreover, S100A8 can induce the activation of MPO, and novel monoclonal antibody against it efficiently prevents lung cancer metastasis (Kinoshita et al., 2019). TRIM29 is a selective regulator of the activation of alveolar macrophages and the production of proinflammatory cytokines in the lungs and is found to mediate lung squamous cell carcinoma cell metastasis by regulating autophagic degradation of E-cadherin.

There are some limitations in this study. First, although we validated the results using external cohorts, the sample size in our cohort is small, reducing the credibility and generalizability of the conclusion. Second, there is no experiment verification of our proposal, for instance, the relationship between model genes and neutrophil. Third, we cannot demonstrate that the affected survival is due to neutrophil population, which requires further *in vivo* analysis.

## Conclusion

Using the immune panel sequencing of our samples, we profiled prognostic immune genes and constructed a risk signature, which confers an excellent and robust predictive power. And this signature is associated with tumor stage, malignant property, and, more importantly, the abundance of neutrophil.

## Data Availability

Publicly available datasets were analyzed in this study. This data can be found here: https://portal.gdc.cancer.gov/ and https://www.ncbi.nlm.nih.gov/geo/. The raw data supporting the conclusions of this article will be available on reasonable request to the corresponding author.

## Supplementary Material

Supplementary Figure S1 Kaplan–Meier analysis of six genes in the training cohort.

Supplementary Figure S2 Kaplan–Meier analysis of six genes in the TCGA and GSE68465 datasets.

Supplementary Table S1 The list of immune panel genes.

Supplementary Table S3 The list of 37 prognostic immune genes.
