# Supplementary figures and images for: An Immune Panel Signature Predicts Prognosis of Lung Adenocarcinoma Patients and Correlates With Immune Microenvironment
Source: Front Cell Dev Biol. 2021 Dec 21;9:797984. doi: 10.3389/fcell.2021.797984 (PMC8725798; doi:10.3389/fcell.2021.797984)

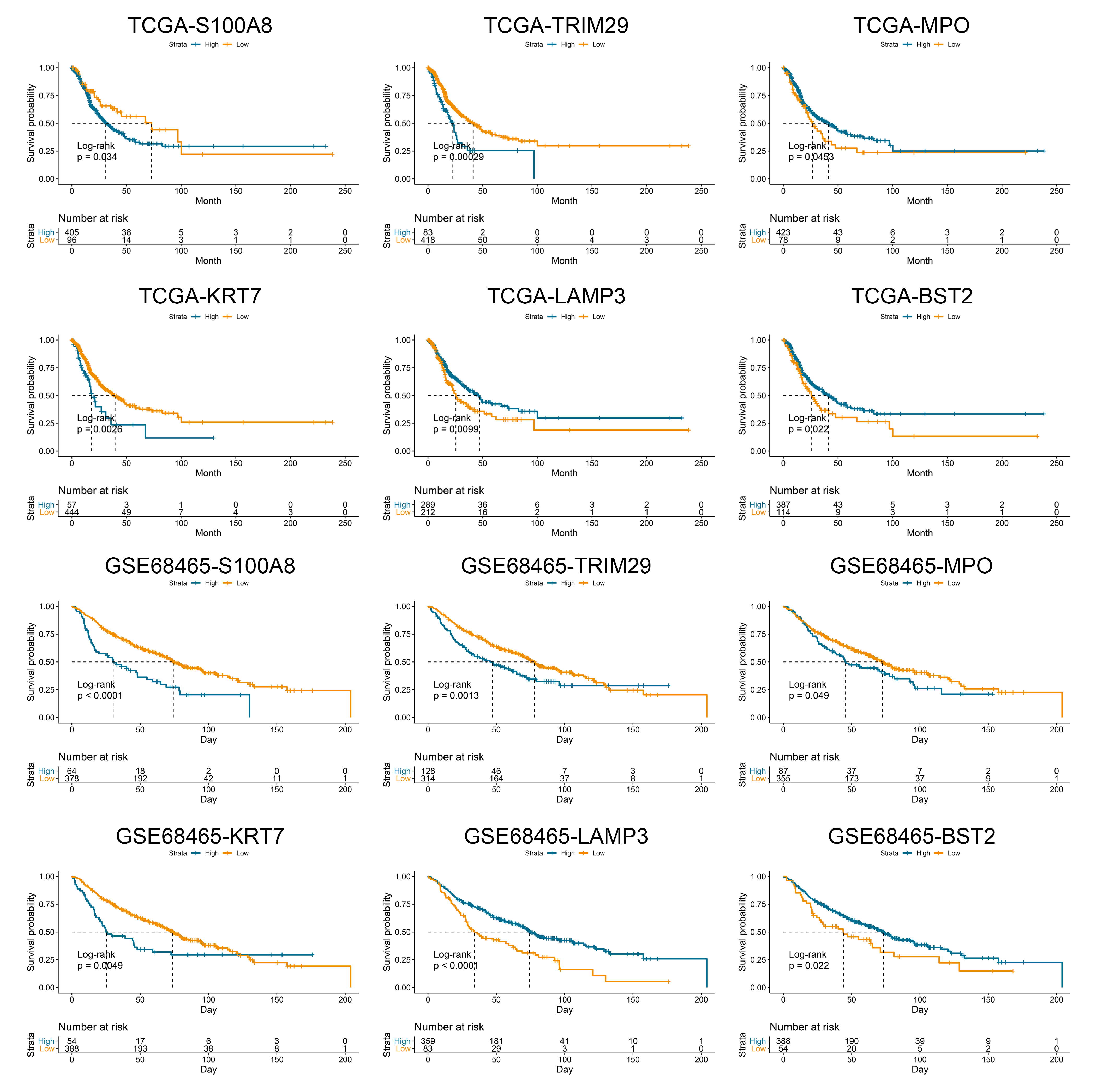

Supplement: Supplementary file 2 [file Image2.TIF]

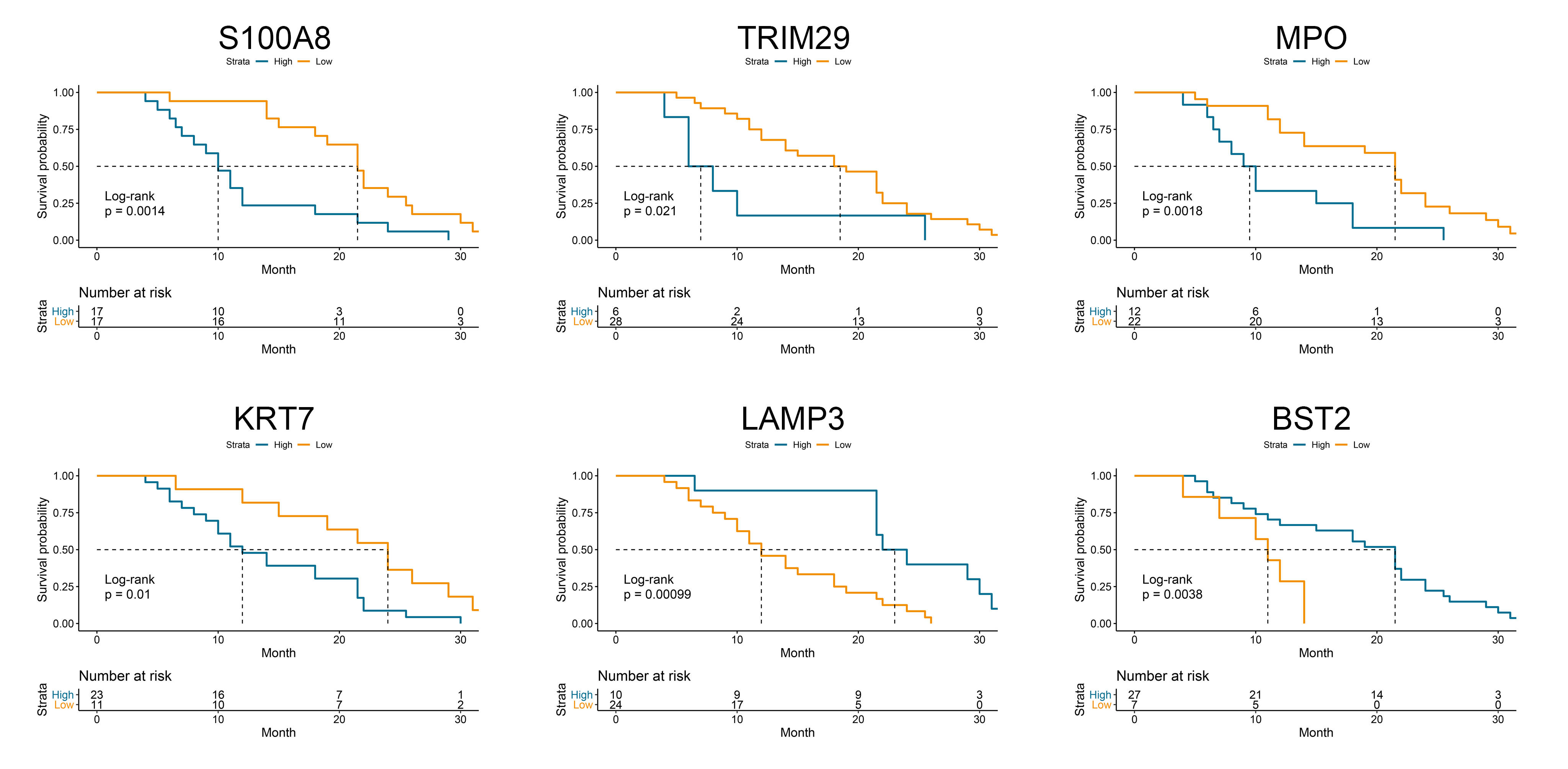

Supplement: Supplementary file 3 [file Image1.TIF]
