# Supplementary material for: An Immune Panel Signature Predicts Prognosis of Lung Adenocarcinoma Patients and Correlates With Immune Microenvironment
Source: Front Cell Dev Biol. 2021 Dec 21;9:797984. doi: 10.3389/fcell.2021.797984 (PMC8725798; doi:10.3389/fcell.2021.797984)
Supplement: Supplementary file 4 [file Table2.docx]

**Table S2. Clinical information of included patients in our cohort (n=34).**

|  | **Total(N=34)** |
| --- | --- |
| **Gender** |  |
| Male | 20 |
| Female | 14 |
| **Age** |  |
| ≤58 | 18 |
| >58 | 16 |
| **N stage** |  |
| N1 | 9 |
| N2 | 25 |
| **T stage** |  |
| T1 | 16 |
| T2 | 13 |
| T3 | 4 |
| T4 | 1 |
| **Differentiation** |  |
| Poorly | 10 |
| Moderate | 13 |
| Well | 11 |
